# A microwave radiation-enhanced Fe–C/persulfate system for the treatment of refractory organic matter from biologically treated landfill leachate†

**DOI:** 10.1039/d1ra04995j

**Published:** 2021-09-02

**Authors:** Yuansi Hu

**Affiliations:** Faculty of Geosciences and Environmental Engineering, Southwest Jiaotong University Chengdu 611756 China 451929326@qq.com

## Abstract

In this study, a microwave (MW) radiation enhanced Fe–C/PS system was used to treat refractory organic matter in biologically-treated landfill leachate. The effects of important influencing factors on the refractory organic matter in biologically treated landfill leachate were explored, and the main reactive oxygen species produced in the system were verified. The mechanism by which humus was degraded was investigated by analyzing effectiveness of organics removal in different systems, and comparative analysis was conducted on the Fe–C materials before and after the reaction. The results showed that degradation capacity and reaction rate of the system could be improved with an increase in the Fe–C/PS dosage and MW power, while initial acidic conditions were also conducive to the degradation of organic matter. Under the conditions of an Fe–C of 1 g L^−1^, PS dosage of 30 mM, MW power of 240 W, and reaction time of 10 min, the UV_254_, TOC, and CN removal efficiencies were 51.48%, 94.56%, and 51.59%, respectively. In the MW/Fe–C/PS system, a large amount of 
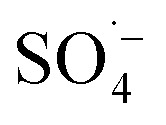
 and a small amount of ˙OH were generated by the thermal activation of PS to remove organic matter. The removal efficiency of organic matter could be further improved *via* the homogeneous catalytic oxidation and heterogeneous adsorption catalytic oxidation of Fe–C materials. In addition, the MW/Fe–C/PS system was effective for removing refractory organic matter from the leachates from four typical treatment systems: DTRO, SAARB, MBR, and NF. The MW/Fe–C/PS system has the potential to be widely applied for the treatment of landfill leachate.

## Abstract 2

A microwave radiation enhanced Fe-C/PS system was used to treat biologically-treated landfill leachate. This process showed wide applicability in treatment of four types of leachates and has a promising potential in landfill leachate treatment.

## Graphical abstract

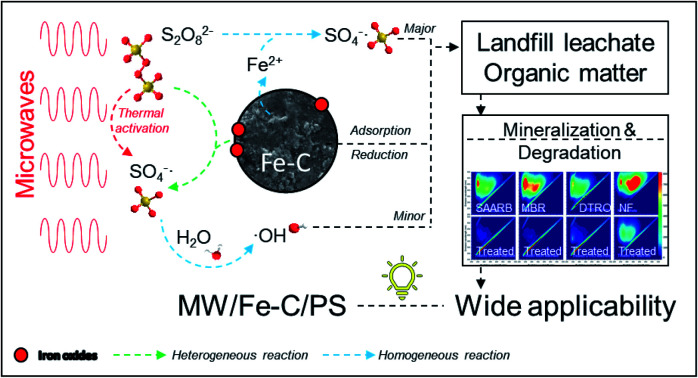

## 1. Introduction

With rapid economic and social development and the acceleration of urbanization, municipal solid waste (MSW) output in China is increasing at an annual rate of 8–10%,^1^ which has inevitably led to the continuous production of landfill leachate. Landfill leachate, as a complex and refractory high-concentration organic wastewater,^2,3^ can cause secondary pollution of the environment, thereby endangering human health and disrupting the balance of ecosystems.^4,5^ Biological and advanced membrane treatments are currently the preferred leachate treatment processes in China,^6–9^ with the typical system being pretreatment + anaerobic + membrane bioreactor (MBR) + nanofiltration (NF) + reverse osmosis (RO). Organic pollutants in the landfill leachate can be effectively removed using this process, but there are broader changes in the water quality and quantity of landfill leachate over time. Landfill leachate typically has the characteristics of a high ammonia nitrogen concentration,^10,11^ high organic matter content,^12,13^ an unbalanced carbon–nitrogen ratio,^14,15^ and poor biodegradability.^16,17^ Traditional biological methods have proven relatively ineffective at removing organic matter from landfill leachate alone, which together with the poor removal of ammonia nitrogen, total nitrogen, and humus has restricted the stability and efficiency of landfill leachate treatment.^8^

The semi-aerobic aged-refuse biofilter (SAARB) is a low-cost and easy-to-operate pretreatment technology for landfill leachate.^18,19^ It uses natural ventilation to form an “aerobic-anoxic-anaerobic” internal environment, enabling the organic matter in leachate to be degraded through “adsorption-exchange-degradation”.^20^ It has a strong removal effect for nitrogenous pollutants, as well as a strong buffering performance on the changes of leachate water quality. However, the effluent from a SAARB (denoted as SAARB leachate) cannot meet the relevant discharge standards due to its high concentration of refractory pollutants, and therefore further advanced treatment needs to be carried out on the SAARB leachate.^21,22^ In contrast, advanced oxidation technology can generate highly chemically reactive free radicals through various physical or chemical reactions (*e.g.*, photoelectric, acoustic, and magnetic reactions), enabling the refractory organic pollutants in wastewater to be rapidly degraded.^23–26^ Persulfate (PS), a strong oxidant that is relatively convenient for storage and transportation, can generate 
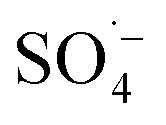
, which has a high redox potential (*E*^0^ = 2.6 V), *via* different activation methods.^27–30^ Its advantage over the ˙OH lies in its greater stability and longer half-life,^31^ and it has therefore become widely applied in the *in situ* remediation of soil and groundwater,^32,33^ as well as the treatment of landfill leachate.^12,34,35^ Persulfate is relatively stable at room temperature with a slow 
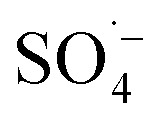
 generation rate, resulting in a low degradation efficiency of organic matter. Many studies have attempted to improve the activation rate of PS and remove refractory organic matter by means of thermal activation,^36^ light activation,^37,38^ alkali activation,^39^ and transition metal activation.^40–42^ Some studies have found that Fe–C materials can effectively activate PS because of their favorable adsorption and catalytic activity.^43,44^ However, when treating wastewater with a high concentration of difficult pollutants and complex components, the contaminated sediment will hinder the effective contact between the heterogeneous catalyst and the oxidant, thereby affecting the treatment effect. Microwaves (MW) radiation provides a means of activation with both thermal and non-thermal effects.^45,46^ They can efficiently promote the activation efficiency of PS and reduce the activation energy and molecular bond strength required for system reaction by virtue of their thermal effects. In addition, their non-thermal effects can not only promote the activation of the oxidant, but also facilitate the regeneration of the filter and maintain the long-lasting surface reactivity. Therefore, the heterogeneous advanced oxidation system has a strong application potential. Few studies have been conducted on the enhancement of an Fe–C/PS system by MW radiation, especially in terms of the removal efficiency and degradation mechanism of refractory organic matter in SAARB leachate.

In this study, an MW-enhanced and Fe–C-activated PS advanced oxidation system (MW/Fe–C/PS) was used to treat refractory organic matter in landfill leachate following treatment with a SAARB. First, the influence of different factors (PS dosage, Fe–C dosage, MW power, and initial pH value) on refractory organic matter in the leachate was investigated. Second, the efficiency of an MW/Fe–C/PS system was analyzed by a comparison of the effectiveness of organic matter removal in different systems and through a three-dimensional (3D) excitation and emission matrix (EEM) analysis. Third, the degradation mechanism of an MW/Fe–C/PS system was analyzed in an alcohol quenching experiment and the physical properties of the Fe–C materials were characterized before and after the reaction. Finally, an MW/Fe–C/PS system was used to treat the leachate resulting from several typical treatments, confirming the applicability and effectiveness of the MW/Fe–C/PS system to treat landfill leachate.

## 2. Materials and methods

### 2.1. Landfill leachates and chemicals

Landfill leachate was collected from a large anaerobic landfill site in southwest China. The leachate was alkaline and dark brown, having the characteristics of a typical aged landfill leachate. The wastewater used in the experiment was the effluent resulting from the pretreatment of landfill leachate by a SAARB. Detailed information of the SAARB was reported by previous studies.^18,19^ The SAARB effluent was light brown, with an initial pH of 8.01, total organic carbon (TOC) content of 190 mg L^−1^, an absorbance at wavelength 254 nm (UV_254_) of 4.39 cm^−1^, and a color number (CN) of 0.1755.

Potassium persulfate, concentrated sulfuric acid (H_2_SO_4_), sodium hydroxide (NaOH), *tert*-butyl alcohol (TBA), and ethyl alcohol (EtOH) were all at analytical grade and were purchased from Chron Chemicals (Chengdu, China). An Fe–C filler was purchased from Puyinworun Environmental Co. Ltd. (Shandong, China). And Fe–C was crushed and screened by passing through a 100-mesh standard sieve for experimental use. A microwave-chemical reactor (MCR-3 type) was purchased from a Yuezhong Equipment Co. Ltd. (Shanghai, China).

### 2.2. Experimental methods

A 100 mL volume of leachate from an aged-refuse biofilter was transferred to a 250 mL round-bottom flask. H_2_SO_4_ and NaOH were used for pH adjustment. A preset amount of Fe–C and potassium persulfate was added and mixed evenly with a stirrer. Then, the mixture was immediately placed into a MW chemical reactor for reaction (10 min). A condensing device was operated during the reaction to reduce the evaporation of the solution. After the reaction was completed, the solution was immediately placed into an ice water bath to cool to room temperature in order to cease the reaction, and then filtered through a 0.45 μm fiber filter membrane. The filtered water sample was tested, and a parallel experiment was then set up repeating the above steps.

### 2.3. Analysis methods

#### 2.3.1. Analysis of water quality indicators

Water quality indicators were analyzed using standard methods.^47^ The pH value was determined by the glass electrode method, TOC was determined by a total organic carbon analyzer (TOC-L CPH CN200, Shimadzu, Kyoto, Japan). The relative content of humus in the SAARB leachate was characterized by UV_254_.^48^ Chromaticity was expressed by the CN, which was calculated as follows:^49,50^1
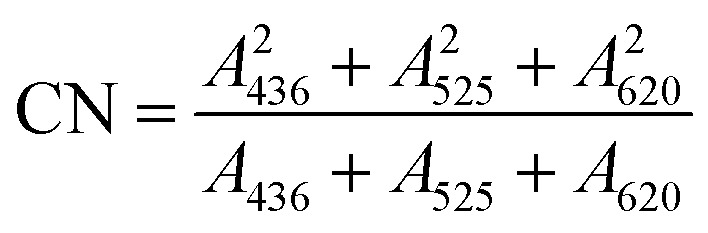


Samples were diluted to a certain multiple with secondary reverse osmosis ultrapure water and then analyzed by a spectrofluorometer (Aqualog-UV-800C, HORIBA, Kyoto, Japan). The fixed excitation wavelength and emission wavelength were both 200–550 nm, with a scanning interval of 5 nm and a scanning speed of 2400 nm min^−1^.

#### 2.3.2. Material characterization

Before and after the reaction, CuKα was used as the radiation source for Fe–C materials. In addition, the tube current was 40 mA and the tube voltage was 30 kV, with a scanning range of 10–80° and a scanning mode of *θ*/2*θ*, continuous scanning. The characterization was conducted using scanning electron microscopy (SEM: 5900LV, Jeol, Tokyo, Japan), X-ray Diffraction (XRD: XD-2, Puxi, Beijing, China), energy dispersive spectroscopy (EDS: Evo18, ZEISS Jena, Germany), and X-ray photoelectron spectroscopy (XPS: Thermo Fisher Scientific, Waltham, MA, USA).

## 3. Results and discussion

### 3.1. Influencing factors

#### 3.1.1. PS dosage

**Fig. 1:**
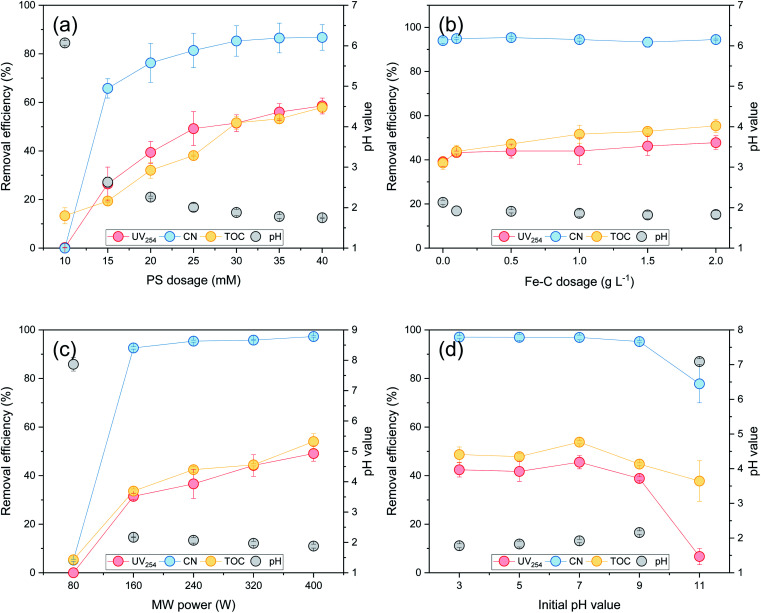
The effect of different experimental conditions on the organic matter removal efficiency and the pH value of the effluent: (a) PS dosage; (b) Fe–C dosage; (c) MW power; (d) initial pH value.

As shown in Fig. 1(a), the UV_254_, TOC, and CN removal efficiencies in the leachate all displayed an upward trend with an increase in the PS dosage. When the PS dosage was 30 mM, the removal efficiencies of UV_254_, TOC, and CN were 51.48%, 51.59%, and 85.31%, respectively. With an increase in the PS dosage, the concentration of reactive oxygen species produced in the system with a high oxidation performance increased. Over time, increasing amounts of 
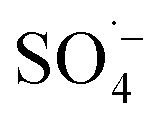
 and ˙OH were produced when PS was activated by contact with the surface of zero-valent iron, iron oxide, and iron oxyhydroxide in the Fe–C system. With an increase in the PS dosage, the solution pH decreased, the concentration of dissolved ferric ions increased, and more 
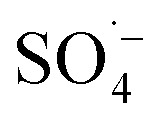
 was generated by the homogeneous activation of PS; thus, increasing the organic matter removal efficiency. However, with a continuous increase in PS dosage, the removal efficiency tended to become stable. This was due to the concentration of organic matter in the system decreasing rapidly, which reduced the probability of contact. Additionally, the generated 
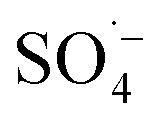
 was easily annihilated by reaction with excess active components, such as S_2_O_8_^2−^,^51,52^ Fe(ii),^53,54^ and 
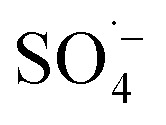
,^55^ which reduced the oxidation utilization rate of PS; thus, restricting the growth of the organic matter removal efficiency.

#### 3.1.2. Fe–C dosage

As shown in Fig. 1(b), the organic matter removal efficiency in the leachate gradually increased with the increase in the Fe–C dosage. When the Fe–C dosage was 2 g L^−1^, the UV_254_ and TOC removal rates increased to 47.77% and 55.40%, respectively. The Fe–C materials had a well-developed porous structure and large specific surface area, which could adsorb and accumulate a small amount of organic matter by van der Waals attraction, and the marginal unsaturated carbon atoms could also provide equally active sites to adsorb nonpolar compounds in wastewater.^56^ An increase in the dosage not only increased the adsorption capacity of organic matter, but also improved the contact probability between organic matter and surface activated free radicals. With an increase in the dosage of Fe–C materials, the contact area involved in PS surface catalysis was larger and the Fe^2+^ concentration released by the reduction of iron increased. This accelerated the generation of 
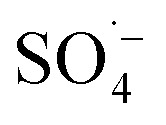
; therefore, reducing the concentration of organic pollutants. Furthermore, the pH value of the effluent decreased with an increase in the Fe–C dosage, which also indicated that the addition of Fe–C could promote the production of 
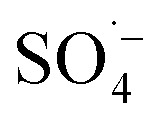
. Therefore, a large number of organic substances could be degraded and converted into acidic intermediate substances, resulting in a decrease in the pH value.

#### 3.1.3. MW power

As shown in Fig. 1(c), the organic matter removal efficiency in the SAARB leachate also increased significantly with an increase in the MW output power. When the power increased from 80 to 400 W, the UV_254_, TOC, and CN removal efficiencies in the SAARB effluent increased by 49.11%, 48.70%, and 92.09%, respectively. This was because an increase in MW power not only facilitated a rapid temperature rise in the system,^30,57^ but also promoted the decomposition of PS to produce 
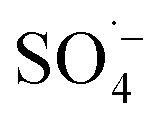
. At the same time, the reaction temperature increased with the increased MW output power. Through these thermal and non-thermal effects, the activation energy and molecular chemical bond strength of the reaction in the system was effectively reduced, the oxidation reaction efficiency was improved, and the organic matter could then be degraded more easily.^58^ However, with a continuous increase in MW output power, organic matter removal increased slowly, which may be attributed to the fact that PS was fully activated within 10 min at a MW power of 240 W, and the oxidation removal of organic matter by free radicals reached a maximum. Therefore, an increase in MW power led to only a slight increase in the organic matter removal efficiency. This was also demonstrated by the rapid decrease in the pH value of the effluent at 240 W, which then tended to remain unchanged.

#### 3.1.4. Initial pH value

As shown in Fig. 1(d), the removal of organic matter by the system under acidic conditions was significantly better than that under alkaline conditions. With an initial pH of 7, the UV_254_, TOC, and CN removal rates in the effluent were 45.54%, 53.80%, and 96.92%, respectively. The electrode reaction was promoted and the corrosion of Fe–C was accelerated under acidic conditions,^59^ and therefore, the oxidation–reduction, electrocoagulation, flocculation, and adsorption processes operated effectively. When the pH value of the reaction system increased, there was a larger amount of ˙OH in the system, which reacted with Fe^2+^/Fe^3+^ to form iron-containing precipitates. The existence of these precipitates in the solution could affect the mass transfer effect and the attack of organic matter by 
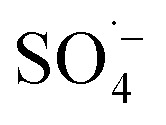
, while PS could be activated efficiently in the system. However, with an increase in the pH value, the probability of the decomposition of PS to 
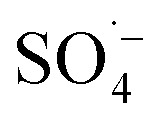
 increased, and the oxidation of organic matter by 
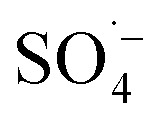
 and ˙OH was further inhibited. Therefore, the removal of organic matter from the SAARB effluent by the MW/Fe–C/PS system was most effective under acidic conditions.

### 3.2. Comparison of the organic removal efficiency of different oxidation systems enhanced by MW

**Fig. 2:**
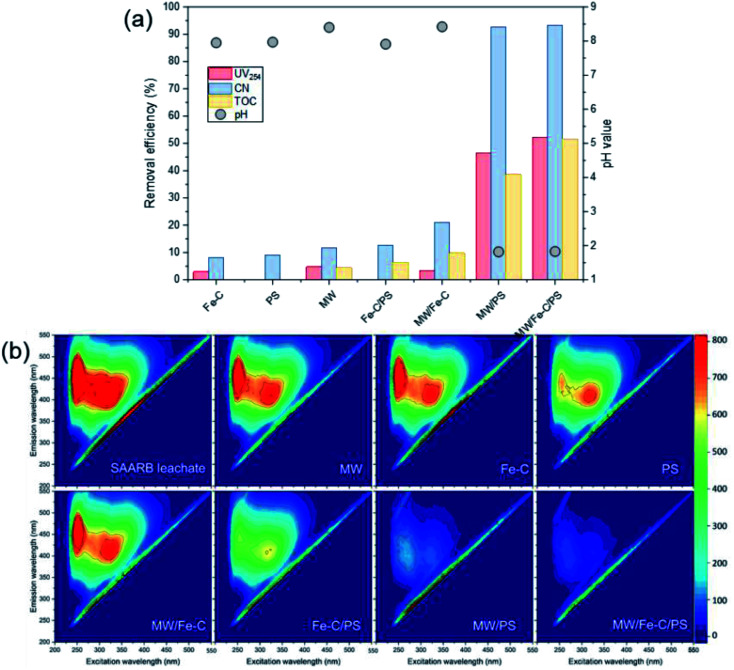
The effects of different treatment systems on the organic matter removal efficiency (a), and three-dimensional EEM spectrum of SAARB leachate treated by different systems (reaction for 10 min under the conditions of an Fe–C concentration of 1 g L^−1^, PS dosage of 30 mM, and MW output of 240 W).

The organic matter removal effect of Fe–C alone, PS alone, and the Fe–C/PS, MW/Fe–C, MW/PS, and MW/Fe–C/PS systems during the treatment of landfill leachate (*i.e.*, SAARB effluent) were compared to determine the mechanism by which humus was degraded in each system. To investigate the activation effect of MW radiation, Fe–C materials, and their combined action on PS and the degradation of humus, an analysis of the removal effects of different leachate treatment systems was conducted using the SAARB effluent. The results are shown in Fig. 2.

#### 3.2.1. Organic removal efficiency

As shown in Fig. 2(a), it was found that Fe–C alone, PS alone, and the Fe–C/PS system resulted in a relatively poor degradation of organic matter in the landfill leachate (*i.e.*, SAARB leachate). In the Fe–C system alone, a small amount of organic matter could be adsorbed by van der Waals attraction because the material had a well-developed porous structure and large specific surface area. The unsaturated carbon atoms at the edges also provided equally active sites to adsorb nonpolar compounds in the wastewater. In the PS system alone, PS did not easily decompose at room temperature,^60^ and only a small amount of 
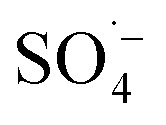
 was generated to degrade some of the organic matter. In the Fe–C/PS system at room temperature, the pH of the solution was still alkaline due to the ineffective hydrolysis of PS, which was higher than the isoelectric point of the catalyst. It was therefore difficult for the catalyst surface to adsorb oxidant on the active sites, and iron ions could not be easily dissolved out. This promoted the activation of PS to a limited extent and slightly improved the organic matter removal efficiency.

Microwaves can reduce the activation energy and molecular bond strength required for the reaction in the system,^58^ and at the same time they increase the reaction temperature, so that some organic matter can be thermally decomposed. However, they cannot directly degrade the humus and other refractory organic matter, and therefore the UV_254_ removal efficiency of the MW system alone was only 4.78%. When MW radiation was introduced into the Fe–C, PS, and Fe–C/PS systems as a means of enhanced activation, the UV_254_ and TOC removal efficiencies in each system increased to different degrees. This can be explained by the fact that MW radiation can cause magnetic materials, such as Fe–C materials, to rapidly accumulate energy and heat up. The chemical bonds in the attached organic matters can be broken, or they can even be mineralized and leave the internal pore space, increasing the potential for physical adsorption. This can also improve the activity of isoactive sites, increase the pace of chemical adsorption, and promote the direct oxidation of organic matter by Fe^0^, surface iron oxide, and Fe^2+^; thus, increasing the removal of organic matter in the Fe–C system to a certain extent. Microwave radiation can rapidly increase the temperature of the system, and thermal activation is an effective way to activate PS to produce 
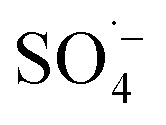
.^57,58^ After heating by MW radiation, the activation rate of PS was greatly improved, and the organic matter removal efficiency in the system was significantly improved. With the addition of MW radiation to the Fe–C/PS system, organic matter removal was further improved. The CN removal efficiency in the leachate reached 93.29%, while the UV_254_ and TOC removal rates reached maximum values of 52.16% and 51.59%, respectively. With the optimization of the Fe–C adsorption performance, more organic matter was adsorbed and degraded, which further improved the organic matter removal efficiency.

#### 3.2.2. 3D-EEM spectra

A number of studies have shown that different types of organic matter are distributed in different fluorescence regions in the 3D-EEM spectrum.^50,61^ As shown in Fig. 2(b), two main emission regions were identified in the leachate analyzed in this study. (1) A fluorescence region in the range of F1 = (Ex/Em = 235–255 nm/410–450 nm) that represented the fulvic acid-like substances in the ultraviolet region, and was mainly caused by low-molecular-weight organic matter with a high fluorescence frequency.^18^ (2) A fluorescence region in the range of F2 = (*E*_x_/*E*_m_ = 310–360 nm/370–450 nm) that represented the fulvic-like substances in the visible light region, and was mainly caused by relatively stable aromatic organic matter with a large molecular weight.^50^

According to Table S1,† the SAARB leachate mainly contained two kinds of substances: fulvic-like substances in the ultraviolet region and fulvic-like substances in the visible light region. The peak intensities of fulvic-like substances in the ultraviolet region and the visible light region in the SAARB leachate were 1398 and 938.1, respectively. Under different process conditions, the peaks of these two regions decreased to varying degrees, and the fulvic-like substances removal efficiencies in the ultraviolet and visible light regions in the MW/Fe–C and MWPS systems were less than 20%, indicating that their removal effect on organic matter in the leachate was weak.

However, the fulvic-like substances removal efficiencies in the ultraviolet and visible light regions by the PS system alone were 52.22% and 21.09%, respectively, indicating that although the activation efficiency of the PS system alone was low at room temperature, the 
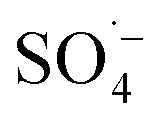
 produced could effectively attack organic matter, breaking the chains and reducing the molecular weight. However, the mineralization (*i.e.*, complete degradation) of organic matter was limited. In the Fe–C/PS system, the activation of PS by Fe–C increased the fulvic-like substances removal efficiencies in the ultraviolet and visible light regions to 61.59% and 34.70%, respectively. In the MW/PS and MW/Fe–C/PS systems, the fulvic-like substances removal efficiencies in the ultraviolet and visible light regions reached 94.48% and 94.43%, respectively, and blue-shift clearly occurred in the fluorescence peak position, indicating that the degree of molecular condensation and molecular weight of dissolved organic matter in the leachate were greatly reduced. Fulvic-like substances in the ultraviolet and visible light regions were effectively degraded into small-molecule organic matter, *i.e.*, the humification degree of organic matter was reduced. This also indicated that the thermal activation of PS was the main process that effectively reduced the humification degree in the MW/Fe–C/PS system. On this basis, the adsorption–oxygen coupling process of Fe–C materials, in which they adsorb and catalyze PS and then oxidize organic matter, can further contribute to the reduction of humification degree of the treated SAARB leachate.

### 3.3. Identification of reactive oxygen species

Reactive oxygen species such as 
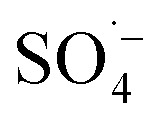
 and ˙OH were produced during the degradation of organic matter by activated PS. The mechanism by which organic matter in the leachate was degraded by the MW/Fe–C/PS system was further investigated, and the major reactive oxygen species in the reaction process were verified. Two radical quenchers (TBA and EtOH) were added to the system in radical quenching experiments under the conditions of an Fe–C concentration of 1 g L^−1^, PS dosage of 30 mM, MW output power of 240 W, and reaction time of 10 min.

**Fig. 3:**
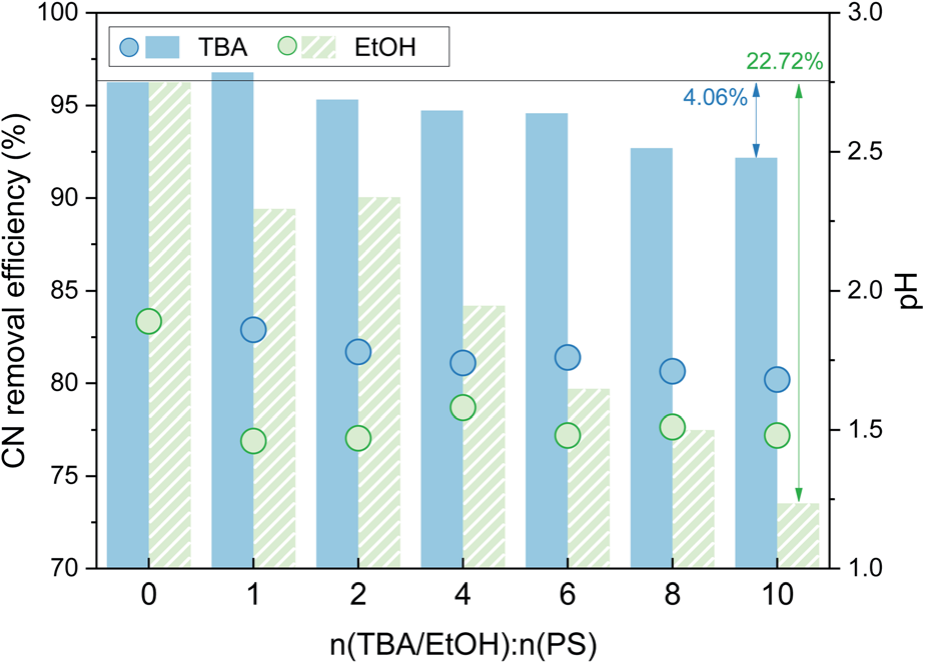
Effects of TBA and EtOH on the CN removal efficiency (*n*(TBA):*n*(PS) = 0, 1, 2, 4, 6, 8, 10; *n*(ETOH):*n*(PS) = 0, 1, 2, 4, 6, 8, 10).

As shown in Fig. 3, with the addition of TBA, the organic matter removal efficiency decreased slightly, and the inhibition effect was not obvious. With the addition of EtOH, there was an obvious decrease in the CN removal efficiency, and when n(EtOH):n(PS) = 10, the removal efficiency decreased by 22.72%. These results indicated that both TBA and EtOH inhibited the removal of organic matter from the SAARB leachate in the MW/Fe–C/PS system to some extent, but the removal of organic matter was only marginally inhibited after adding TBA. The addition of EtOH rapidly inhibited the degradation of organic matter in leachate in the oxidation system, indicating that EtOH had a stronger inhibition effect on the system than TBA.^58^ Generally, TBA is often used as a trapping agent for ˙OH, while EtOH is often used as a trapping agent for both ˙OH and 
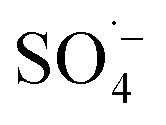
,^15,52^ proving that 
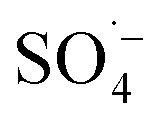
 and ˙OH existed simultaneously in the MW/Fe–C/PS system. The removal of organic matter was predominantly attributed to 
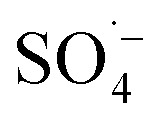
 and was supplemented by ˙OH, indicating that Fe–C combined with MW radiation can effectively activate PS to produce 
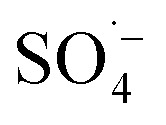
.

### 3.4. Transformation of Fe–C materials in the MW/Fe–C/PS system

**Fig. 4:**
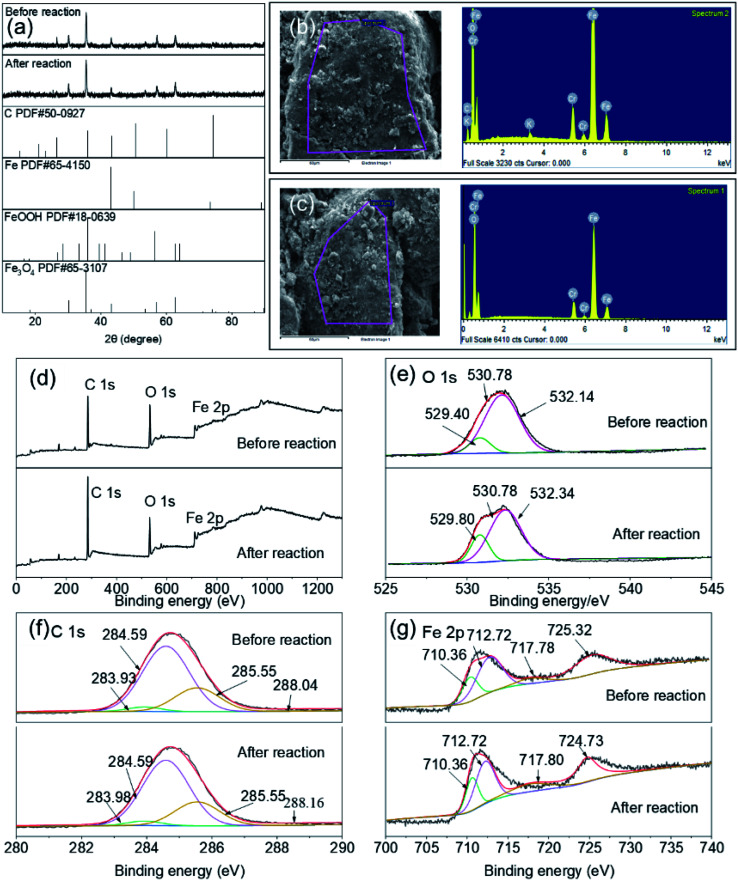
The XRD (a), SEM-EDS (b and c), and XPS (d–g) patterns of Fe–C materials before and after the reaction.

The Fe–C materials were characterized by XRD, SEM, EDS, and XPS before and after the reaction to investigate property changes of Fe–C materials, as well as the morphology, species, and relative content of each element in the Fe–C materials before and after the reaction. Results are shown in Fig. 4.

#### 3.4.1. XRD analysis

Fig. 4(a) shows the XRD patterns of the Fe–C materials before and after the reaction. From a comparison with powder diffraction file (PDF) standards, the 2θ peak coincided with Fe(PDF#65-5150) at 43.13° and 49.96°, and with C(PDF#50-0927) at 26.52°, 36.03°, and 43.32°, indicating that there was a large amount of elemental Fe and C prior to the Fe–C reaction. At the same time, there were obvious 2*θ* peaks at 30.16°, 35.51°, 57.13°, 62.70°, and 89.93° before the reaction, indicating the presence of iron oxide and iron oxyhydroxide in the Fe–C materials. Many typical peaks were still present in the Fe–C materials after the reaction, indicating that the Fe–C materials still contained elemental Fe and C after the reaction. In addition, the 2*θ* peaks at 30.16°, 35.51°, and 57.13° increased, indicating that in this system, Fe^0^ formed iron oxides (such as Fe_3_O_4_) after the reaction.

#### 3.4.2. SEM-EDS analysis

Fig. 4(b and c) show the SEM and EDS patterns of Fe–C materials before and after the reaction, which enabled the changes in the surface morphology of Fe–C materials to be characterized. It can be seen from Fig. 4(b) that before the treatment of the SAARB leachate, the surface of the Fe–C material was relatively rough, the grain sizes were uneven and scattered, and there was a specific pore structure. The EDS pattern revealed a large Fe, O, and C content near the 0–2 keV interval, indicating that iron oxide was the main component in the large grains. There was a large Fe content in the 6–8 keV interval, indicating that Fe in small grains was mainly attached to the surface of C. It can be seen from Fig. 4(c) that Fe–C material could easily form iron corrosion products in an aerobic environment. From the peaks of C, O, and Fe in the EDS pattern and the decrease in their content after the reaction, it was inferred that as the reaction progressed, part of the Fe was detached from the surface of the main crystal phase, and iron oxide and elemental Fe on the surface of the material were electrochemically corroded.

#### 3.4.3. XPS analysis

Fig. 4(d) shows the XPS full scanning spectrum of Fe–C materials before and after the reaction, enabling the morphology, species, and relative content of various elements in the Fe–C materials to be investigated. It can be seen from the figure that there were three typical characteristic peaks in the material, *i.e.*, C 1s, O 1s, and Fe 2p, indicating that the Fe–C materials were mainly composed of C, O, and Fe. By analyzing and comparing the peak patterns before and after the reaction of Fe–C materials it was found that the peak values of C and Fe displayed an upward trend, while the peak values of O displayed a downward trend. To better analyze the existing state, species, and relative contents of C, O, and Fe, O 1s, C 1s, and Fe 2p high resolution spectra of Fe–C materials were further analyzed. The results are illustrated in Fig. 4(e–g).

It can be seen from the peak diagram of O 1s that the binding energies corresponded to O^2^˙^−^, ˙OH, and H_2_O at 529.40, 530.78, and 532.14 eV, respectively. As can be seen from Table S2,† the relative contents of O^2^˙^−^ and ˙OH increased to 1.88% and 23.15%, respectively, after the reaction, implying that C played a role in adsorption and skeleton support during the removal of organic matter. The Fe and iron oxides loaded on the C skeleton played a leading role and could effectively participate in the reaction to form Fe(iii), which could complex with organic functional groups on the carbon surface to achieve the removal of pollutants.

It can be seen from the C 1s peak diagram that the binding energies at 283.93, 284.59, 285.55, and 288.04 eV corresponded to C–C, C–O, C=O, and O=C=O bonds, respectively. It can be seen from Table S2† that the contents of the C–C, C=O and O=C=O bonds were reduced after the reaction, indicating that the Fe–C materials could destroy the oxygen-containing functional groups after the reaction, and the C–C content was reduced from 21.96% to 8.68%, indicating that C has a strong adsorption effect on organic matter and participates in the removal of organic matter during the reaction. In addition, the relative content of C–O increased from 41.32% to 66.18% due to the adsorption of unsaturated bonds.

It can be seen from the Fe 2p peak diagram that the binding energies at 710.36, 712.72, 717.78, and 723.32 corresponded to Fe^0^, Fe(ii), Fe(iii), and Fe_3_O_4_, respectively. According to Table S2,† the relative contents of Fe(ii) and Fe_3_O_4_ both decreased, while the contents of both Fe^0^ and Fe(iii) increased, indicating that Fe(ii) could destroy the molecular structure of organic matter in this reaction system, reducing macromolecular organic matter to small molecular organic matter and transforming it into Fe^0^ and Fe(iii). The relative content of Fe_3_O_4_ displayed a decreasing trend before and after the reaction, which indicated that the Fe(iii) contained in the reaction also participated in the formation of organic complex precipitates and formed a transformation chain of iron with different valence states. Therefore, the Fe–C materials after the reaction still had a certain oxidation–reduction ability.

### 3.5. Wide applicability

Landfill leachate is a high-concentration wastewater containing a variety of organic and inorganic substances. Treatment techniques need to be buffered against changes in the composition and concentration of contaminants given that the composition of different leachates varies greatly. The MW/Fe–C/PS system, as an advanced oxidation treatment technology with 
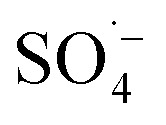
 as the main reactive oxygen species, could play a unique role in effectively degrading most pollutants and adapting to the treatment needs of various types of wastewater, with a range of different compositions, by adjusting the reaction conditions to control the action intensity of oxidants. The effect of this system on the removal of refractory organic matter from different types of landfill leachate was studied, and a reliability analysis was conducted to determine the effectiveness of an MW-Fe–C/PS system for the treatment of landfill leachate containing different pollutants and other high concentration organic wastewaters.

Leachates from four typical treatment systems, disk tube-reverse osmosis (DTRO), SAARB, MBR, and NF, were used as the research objects. For the SAARB and MBR, leachate effluents were treated by the SAARB and a two-stage biological nitrogen removal system, respectively, and the biodegradability was substantially reduced and the proportion of refractory organic matter was high. The DTRO and NF leachates were the concentrated (retentate) solutions of biologically treated effluent that were produced after treatment by DTRO or NF membranes, respectively. The concentration of refractory organic matter was further enriched, and the concentrations of organic matter in each leachate were different (see Table 1 for details). Under the conditions of an Fe–C concentration of 1 g L^−1^, MW output power of 240 W, and reaction time of 10 min, the PS dosage (oxidant) was varied (PS_DTRO_ = 10 mM; PS_SAARB_ = 30 mM; PS_MBR_ = 60 mM; PS_NF_ = 180 mM), and the MW/Fe–C/PS system was used to treat each of the four leachates.

**Table 1:** Effluent quality of four typical landfill leachates

Leachate	CN (cu)	UV_254_ (cm^−1^)	TOC (mg L^−1^)
SAARB leachate	0.194	3.96	207.025
MBR leachate	0.371	5.45	288.75
DTRO leachate	0.074	1.38	154.625
NF leachate	1.499	24.01	2459.75

#### 3.5.1. Organic matter removal performance

**Fig. 5:**
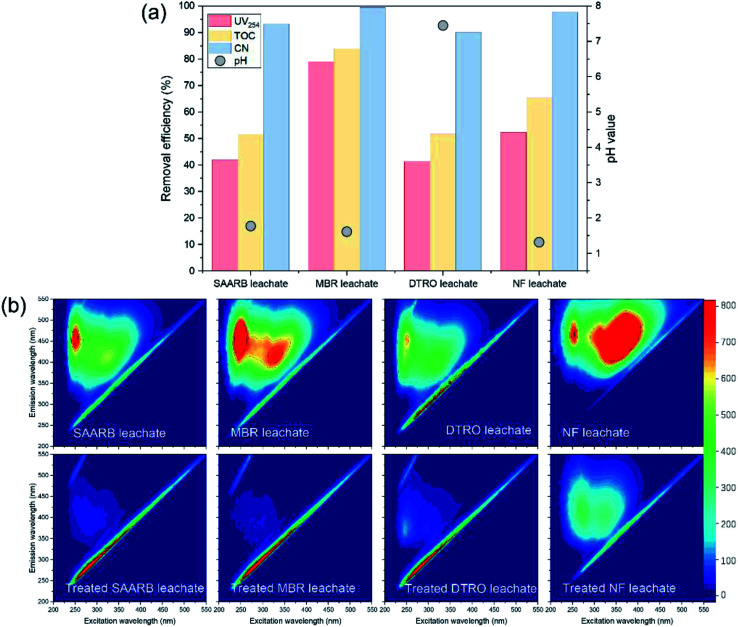
Performance of MW/Fe–C/PS system in SAARB, MBR, DTRO, and NF leachates treatment (a), and three-dimensional EEM spectrum of different water samples treated by an MW/Fe–C/PS system.

The organic matter removals from the four leachates treated with the MW/Fe–C/PS system and the humus degradation revealed by 3D-EEM are shown in Fig. 5. When the MW/Fe–C/PS system was used to treat the DTRO, SAARB, MBR, and NF leachates, as shown in Fig. 5(a), it was found that organic matter in the system was both removed effectively. The MW/Fe–C/PS system removed more than 40% of the UV_254_, 50% of the TOC, and more than 90% of the CN from the DTRO, SAARB, MBR, and NF leachates. The best results were obtained for the MBR leachate, with UV_254_, TOC, and CN removal efficiencies of 78.90%, 83.94%, and 99.41%, respectively. The results confirmed that the MW/Fe–C/PS system could decompose organic pollutants in various landfill leachates in a stable and efficient manner.

The removal performance of MW/Fe–C/PS system on four leachates containing substances with different humification degree was further evaluated. As shown in Fig. 5(b), the four leachates from SAARB, MBR, DTRO, and NF had obvious peaks at F1 and F2, and the relative intensity of these two luminescent regions was significantly different in different water samples. As shown in Table S3,† the relative intensity ranges of the F1 and F2 peaks for the four leachates were 659–1266 and 460.5–1533 respectively, among which the maximum relative intensities of F1 and F2 were 1266 (MBR) and 1533 (NF), respectively. It is generally believed that the lower the humification degree of organic matter, the lower the benzene-ring content, the lower the condensation degree of aromatic compounds, and the shorter the fluorescence excitation corresponding to the peak of humic-like substances. All four leachates contained fulvic-like substances in the ultraviolet region and fulvic-like substances in the visible light region, but there were differences in the composition and concentration of benzene-ring and aromatic refractory pollutants. The relative intensity of these two fluorescent regions in the four leachates treated by the MW/Fe–C/PS system decreased to a large extent, and the peak position of the fluorescence peaks occurred blue-shift. The removal efficiencies of fulvic-like substances in the ultraviolet region and fulvic-like substances in the visible light region reached over 90% for the SAARB and MBR leachates, which indicated that the degree of molecular condensation and molecular weight of aromatic compounds in the leachates decreased greatly, while the degree of humification had increased. These results indicate that the MW/Fe–C/PS system has a wide practicability and feasibility for the treatment of landfill leachate.

### 3.6. Proposed reaction mechanism of MW/Fe–C/PS system

**Fig. 6:**
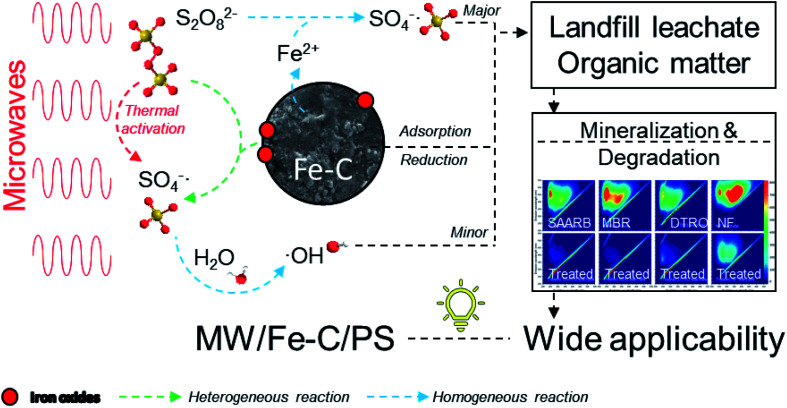
Proposed reaction mechanism of MW/Fe–C/PS system.

The reaction mechanism was proposed and illustrated in Fig. 6. In the MW/Fe–C/PS system, the system temperature rose rapidly under the action of MW radiation and the PS was thermally activated. At the same time, Fe–C itself has a certain adsorption capacity, reduction capability, and catalytic property, which can activate PS on the surface of the catalyst (heterogeneous catalysis) and in the solution (homogeneous catalysis). Both the 
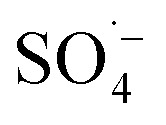
 generated by PS activation and the ˙OH generated by its chain reaction were used to remove organic matter.

#### 3.6.1. Thermal activation of PS

Under the action of MW radiation, the temperature of the system rose rapidly. After the PS adsorbed on the catalyst surface and solution absorbed heat energy, the O–O bond was broken to form 
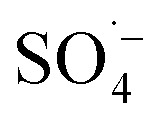
, which can react with H_2_O/OH^−^ to further generate a small amount of HO˙. Both 
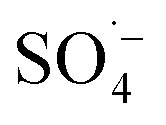
 and HO˙ are highly oxidative reactive oxygen species with an unsaturated electronic structure, which can react with the organic matter adsorbed on the catalyst surface or the organic matter obtained by rapid molecular movement in the solution to remove the organic matter.

#### 3.6.2. Reduction reaction of Fe–C

There is a large amount of elemental iron in Fe–C, which has the characteristics of a high electronegativity and strong reducing ability. After the PS hydrolysis, the solution was acidic. On the one hand, Fe^0^ can directly provide electrons to generate active hydrogen, and the active hydrogen with a strong reducing effect transfers electrons to the organic matter in the SAARB leachate. This can destroy the chromophore or auxochrome groups, decompose the macromolecular aromatic organic matter into small molecular acidic intermediate products, and improve the biodegradability of wastewater to a certain extent. In addition, it can continue to provide electrons to the Fe^3+^ produced by the oxidation reaction. On the other hand, Fe^0^ can react with O_2_, H_2_O, and H^+^, and the Fe^2+^ that is produced has the same reducing effect and can directly participate in the decomposition of organic matter.

#### 3.6.3. Adsorption and precipitation of Fe–C

The reaction system contained a certain amount of carbon and a large amount of iron, iron oxides, and hydroxyl ferric oxides. These substances can fix the enriched organic matter in the pores and on the surface by van der Waals forces or the active sites of unsaturated bonds, and quickly degrade organic matter under the action of MW to restore adsorption spaces. Adsorption can cause the organic matter in the system to rapidly contact the ˙OH generated by activation, at which point it can be degraded.

#### 3.6.4. Heterogeneous catalysis of Fe–C

In the Fe–C system, the surface active sites such as iron oxide, iron oxyhydroxide, and marginal unsaturated carbon atoms added during the reaction process can catalyze PS to produce a large amount of 
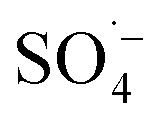
 and a small amount of HO˙, which preferentially attack the organic matter adsorbed on the surface of the material, degrading and desorbing it into the solution. This process then continues to adsorb the organic matter in the solution after the adsorption site is vacated for the next adsorption oxidation.

#### 3.6.5. Homogeneous catalysis of Fe–C

In the Fe–C system, the Fe^2+^ produced by the reduction of Fe^0^ under acidic conditions precipitates as the main crystal, and the free Fe^2+^ in the system loses its electrons, which promotes the activation of PS to produce 
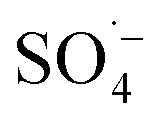
. With the chain reaction of 
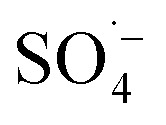
, a small amount of ˙OH is formed and further contact is made with the fast moving organic molecules in the high temperature solution. The organic matter was degraded and removed by electron transfer, hydrogen extraction, and an addition reaction.

## 4. Conclusions

Landfill leachate is a high-concentration organic wastewater that is difficult to treat and harmful to the environment. Leachate treated by the SAARB still cannot meet the relevant discharge standards, and therefore, further advanced treatment is needed. In this study, MW-enhanced and Fe–C-activated co-persulfate were used to treat refractory organic matter in leachate (*i.e.*, SAARB leachate). The following results were obtained.

(1) An increase in the PS dosage enabled the system to produce more 
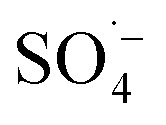
; thus, degrading organic matter. The use of Fe–C benefits the system due to its strong adsorption performance and release of Fe^2+^ to catalyze PS, producing highly active 
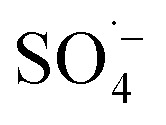
. With an increase in MW power, the system was rapidly heated *via* a thermal effect, while the non-thermal effect of MW radiation made the pollutants more vulnerable to attack by 
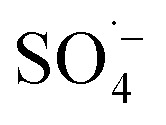
; thus, improving the reaction efficiency. The initial conditions were acidic, which were beneficial for the degradation of organic matter.

(2) Under the conditions of an Fe–C concentration of 1 g L^−1^, PS dosage of 30 mM, MW output power of 240 W, and reaction time of 10 min, the UV_254_, TOC, and CN removal efficiencies from leachate were 51.48%, 94.56%, and 51.59%, respectively.

(3) In the MW/Fe–C/PS system, the organic matter in the SAARB leachate was mainly degraded by the high-efficiency activation of PS by the thermal effect of MW radiation and the homogenous and heterogeneous activation of iron species, while the adsorption–oxidation coupling system of Fe–C further improved the degradation efficiency of organic matter.

(4) The MW/Fe–C/PS system has a strong removal effect on the refractory organic matter from the SAARB, MBR, DTRO, and NF leachates. Therefore, the MW/Fe–C/PS system has the potential to be widely applied for the treatment of landfill leachate.

## Conflicts of interest

There are no conflicts to declare.

## Supplementary Material

RA-011-D1RA04995J-s001
