# Supplementary material for: A microwave radiation-enhanced Fe–C/persulfate system for the treatment of refractory organic matter from biologically treated landfill leachate
Source: RSC Adv. 2021 Sep 2;11(47):29620–31. doi: 10.1039/d1ra04995j (PMC9040904; doi:10.1039/d1ra04995j)
Supplement: RA-011-D1RA04995J-s001 [file RA-011-D1RA04995J-s001.pdf]

Electronic Supplementary Material (ESI) for RSC Advances.
This journal is © The Royal Society of Chemistry 2021

Supporting information for

A Microwave Radiation-enhanced Fe-C/Persulfate System for the Treatment of Refractory Organic Matter from Biologically Treated Landfill Leachate

Yuansi Hu*

Faculty of Geosciences and Environmental Engineering, Southwest Jiaotong University, Chengdu, 611756, China

*Corresponding author, Email: 451929326@qq.com (Yuansi Hu)

*Submitted to **RSC Advances***

Table Captions

Table S1. Comparison of the 3D-EEM peak positions and values for SAARB leachate treated by different systems.

Table S2. The XPS peak fitting results for Fe-C before and after the reaction.

Table S3. Comparison of the fluorescence peak positions and peak intensities of different water samples treated by an MW/Fe-C/PS system.

Table S1. Comparison of the 3D-EEM peak positions and values for SAARB leachate treated by different systems.

System	F1	Intensity	Removal	F2	Intensity	Removal
SAARB leachate	255/455	1398	/	320/410	938.1	/
MW	250/455	1220	12.73%	325/410	816.7	12.94%
Fe-C	250/455	1213	13.23%	320/415	769.8	17.94%
PS	255/440	667.9	52.22%	315/410	740.3	21.09%
MW/Fe-C	250/460	1176	15.88%	325/410	796.2	15.13%
Fe-C/PS	250/440	537	61.59%	315/410	612.6	34.70%
MW/PS	255/415	122.2	91.26%	325/410	93.44	90.04%
MW/Fe-C/PS	245/420	70.16	94.98%	325/405	52.23	94.43%

Table S2. The XPS peak fitting results for Fe-C before and after the reaction.

Type	Peak	Binding energy (eV)	Peak area		Relative percentage (%)	
			Before	After	Before	After
O 1S	O^{2-}	529.4	1245.74	1407.312	1.5	1.88
	OH^{-}	530.78	11389.04	17294.12	13.75	23.15
	H_2O	532.14	70217.52	56009.96	84.75	74.97
C 1S	C-C	283.93	17672.3	8869.372	21.96	8.68
	C-O	284.59	33251.28	67658.7	41.32	66.18
	C=O	285.55	26731.72	23992.34	33.21	23.47
	O=C=O	288.04	2826.106	1709.019	3.51	1.67
Fe 2p	Fe^0	710.36	7979.074	8961.739	16.77	18.35
	Fe(II)	712.72	18865.06	17150.33	39.66	35.12
	Fe(III)	717.78	3159.218	7593.287	6.64	15.55
	Fe_3O_4	725.32	17564.01	15130.84	36.92	30.98

Table S3. Comparison of the fluorescence peak positions and peak intensities of different water samples treated by an MW/Fe-C/PS system.

System	F1	Intensity	Removal	F2	Intensity	Removal
SAARB	250/455	860	/	330/415	542.2	/
MBR	250/455	1266	/	325/410	776.1	/
DTRO	250/455	659	/	320/400	460.5	/
NF	255/450	731.5	/	360/450	1533	/
SAARB	255/415	43.13	94.98%	310/410	38.18	92.96%
MBR	250/425	24.76	98.04%	310/405	31.56	95.93%
DTRO	250/410	81.37	87.65%	310/370	48.45	89.48%
NF	255/415	256.9	64.88%	325/405	293.9	80.83%
